# Environmental stressors may cause equine herpesvirus reactivation in captive Grévy’s zebras (*Equus grevyi*)

**DOI:** 10.7717/peerj.5422

**Published:** 2018-08-22

**Authors:** Peter A. Seeber, Benoît Quintard, Florian Sicks, Martin Dehnhard, Alex D. Greenwood, Mathias Franz

**Affiliations:** 1 Department of Wildlife Diseases, Leibniz Institute for Zoo and Wildlife Research, Berlin, Germany; 2 Parc Zoologique & Botanique de Mulhouse, Mulhouse, France; 3 Tierpark Berlin, Berlin, Germany; 4 Department of Reproduction Biology, Leibniz Institute for Zoo and Wildlife Research, Berlin, Germany; 5 Department of Veterinary Medicine, Freie Universität Berlin, Berlin, Germany

**Keywords:** EHV, Latent infection, Reactivation, Fecal glucocorticoids

## Abstract

Equine Herpesviruses (EHV) are common and often latent pathogens of equids which can cause fatalities when transmitted to non-equids. Stress and elevated glucocorticoids have been associated with EHV reactivation in domestic horses, but little is known about the correlation between stress and viral reactivation in wild equids. We investigated the effect of an environmental stressor (social group restructuring following a translocation event) on EHV reactivation in captive Grévy’s zebras (*Equus grevyi*). A mare was translocated by road transport from Zoo Mulhouse, France, to join a resident group of three mares in Tierpark Berlin, Germany. We used an indirect sampling method to assess the frequency of EHV shedding for 14 days immediately after the translocation event (termed the ‘experimental period’). The results were compared with those from two control periods, one preceding and one subsequent to the experimental period. In addition, we measured fecal glucocorticoid metabolite (fGCM) concentrations daily in all individuals from 6 days before, to 14 days after translocation. We found significantly higher EHV shedding frequencies during the experimental period, compared to each of the two control periods. All animals showed significantly elevated fGCM concentrations, compared to fGCM levels before translocation. Finally, we found that an increase in fGCM concentration was significantly associated with an increased likelihood of EHV shedding. Although the small number of animals in the study limits the conclusions that can be drawn from the study, taken together, our results support the hypothesis that environmental stressors induce viral reactivation in wild equids. Our results suggest that potentials stressors such as group restructuring and translocation should be considered in the management of zoological collections to reduce the risk of fatal EHV infections in novel hosts. Moreover, environmental stressors may play an important role in EHV reactivation and spread in wild equid populations.

## Introduction

Equine Herpesviruses (EHV) are common pathogens in equid populations and are responsible for considerable economic losses (Lunn et al., 2009; Dunowska, 2014a). EHVs can be subdivided into two subfamilies within the family of *Herpesviridae*, alpha- and gamma-herpesviridae (Davison, 2010). There is a wide spectrum of clinical manifestation with pathogenicity differing substantially between and among virus strains (Chowdhury, Kubin & Ludwig, 1986; Van de Walle et al., 2009; Azab & Osterrieder, 2012). In equids, acute EHV infection can manifest as fever, nasal discharge, inflammation of the upper respiratory tract, degenerative neurological disease, lymphadenopathy, conjunctivitis, coital exanthema, multinodular pneumonia, and abortion (Patel & Heldens, 2005; Fortier et al., 2010; Dunowska, 2014b; Marenzoni et al., 2015).

EHVs can infect a wide range of host species other than equids, with novel hosts including Thomson’s gazelle (*Eudorcas thmomsonii*; Fukushi et al., 1997), giraffe (*Giraffa camelopardalis*; Schrenzel et al., 2008), llamas and alpacas (*Lama glama*, *Vicugna pacos* respectively, Rebhun et al., 1988), polar bears (*Ursus maritimus*; Greenwood et al., 2012), brown bears (*Ursus americanus*; Wohlsein et al., 2011), and guinea pigs (*Cavia porcellus*; Wohlsein et al., 2011). In these novel hosts, EHV infections are typically more severe than in equids and often fatal. Clinical signs in novel host species can manifest as respiratory disease, retinitis, abortion, neonatal death and myeloencephalopathy (Greenwood et al., 2012; Ma, Azab & Osterrieder, 2013; Abdelgawad et al., 2014).

Transmission of EHV occurs only during phases of acute viral replication when the virus is actively shed into the environment by its host (Dunowska, 2014b; Marenzoni et al., 2015). However, all herpesviruses share the ability to establish latent infections which can last for the lifetime of their hosts (Carter, Wise & Flores, 2006). The time span of viral latency is characterized by absence of significant viral replication and minimal viral gene expression, despite presence of the viral genome in the nucleus of the infected cell (White, Suzanne Beard & Barton, 2012; Reese, 2016). In contrast to human herpesviruses, reactivation of latent EHV infections and the associated physiological conditions are not well understood, but it is generally assumed that exposure to environmental stressors play a major role in the reactivation of latent viral infections (Padgett et al., 1998; Glaser & Kiecolt-Glaser, 2005; Dunowska, 2014b; Sebastiano et al., 2017).

In horses, the stress response to transportation has been linked to EHV reactivation, which in turn can trigger virus transmission and related clinical outbreaks of novel strains (Pusterla et al., 2009; Barbić et al., 2012; Badenhorst et al., 2015). Handling and transport are the potential source of stress for the translocated individuals. In addition social stress after introduction of a new individual might affect all animals concerned. For example in domestic horses, changes in their social environment can elicit a physiological stress response (Alexander & Irvine, 1998; York & Schulte, 2014; Nuñez et al., 2014).

Stress responses to transportation and to changes in the social environment might also contribute to EHV reactivation and transmission in other equid species. Changes in the social environment following translocation events typically induce a substantial stress response in various wildlife species, including equids (Franceschini et al., 2008; Schmidt et al., 2010; Dickens, Delehanty & Michael Romero, 2010; Vick et al., 2012). However, all available information on the effect of stress responses on EHV reactivation and shedding in equids originates from studies on domestic horses or ponies. Whether in other equids, translocation and changes in the social environment elicits stress responses that lead to EHV reactivation and transmission is unclear.

We conducted a study on the effects of social group restructuring following a translocation event, in captive Grévy’s zebras (*Equus grevyi* Oustalet, 1882). First, we tested whether the frequency of EHV shedding was elevated in the experimental time period immediately after the translocation event, compared to two control periods (before and after the experimental period). Secondly, we assessed whether the translocation event and subsequent social re-structuring induce physiological stress responses. We tested whether there is a direct correspondence between levels of physiological stress responses and EHV shedding for all animals. Longitudinal data was collected on the severity of the physiological stress response by measuring glucocorticoid (GC) metabolites in fecal samples (Ganswindt et al., 2012). Fecal glucocorticoid metabolites (fGCM) have the considerable advantage that they reflect cumulative secretion and elimination of GC’s over most of the gut passage, as opposed to time specific GC measurements in blood (Sheriff et al., 2011; Kersey & Dehnhard, 2014). Finally, we genotyped EHV strains shed by different individuals to assess potential inter-individual EHV transmission as a result of stress-induced viral shedding. The small sample size and limited access to control conditions constrains the conclusions that can be drawn. However, the study may provide an indication of general patterns of stress and EHV shedding in captive zebras.

## Materials & Methods

### Ethics statement

This study was approved by the Internal Ethics Committee of the Leibniz Institute for Zoo and Wildlife Research (Approval number 2017-02-01), and was approved by the two institutions housing the animals, Zoo Mulhouse and Tierpark Berlin.

### Study animals and study set-up

We investigated EHV shedding and measured fGCM concentrations in four captive mature Grévy’s zebra mares which at the time of this study were neither pregnant nor lactating. One of these mares (“Ekwe”) was initially housed at Zoo Mulhouse, France, and during the course of this study was translocated to Tierpark Berlin (a roughly 850 km distance by road transport) where she joined a resident group of three mares (“Franzi”, “Kianga”, “Zawadi”). No sedative was administered to the transported individual.

For the first four days after arrival in Berlin, the translocated mare was kept in an enclosure that was separated by a wire fence from the enclosure of the resident mares. From day 5 to 9 post-translocation the four mares shared an enclosure for the first time, but were separated again from day 10 to 26 to allow for additional feeding of roughage to the new mare. Based on a previous study the three mares in Tierpark Berlin were known to be latently infected with at least two EHV strains (EHV-1, and *Equus zebra*-Herpesvirus) (Seeber et al., 2017). The EHV infection status of the new mare prior to translocation was unknown.

**Figure 1:**
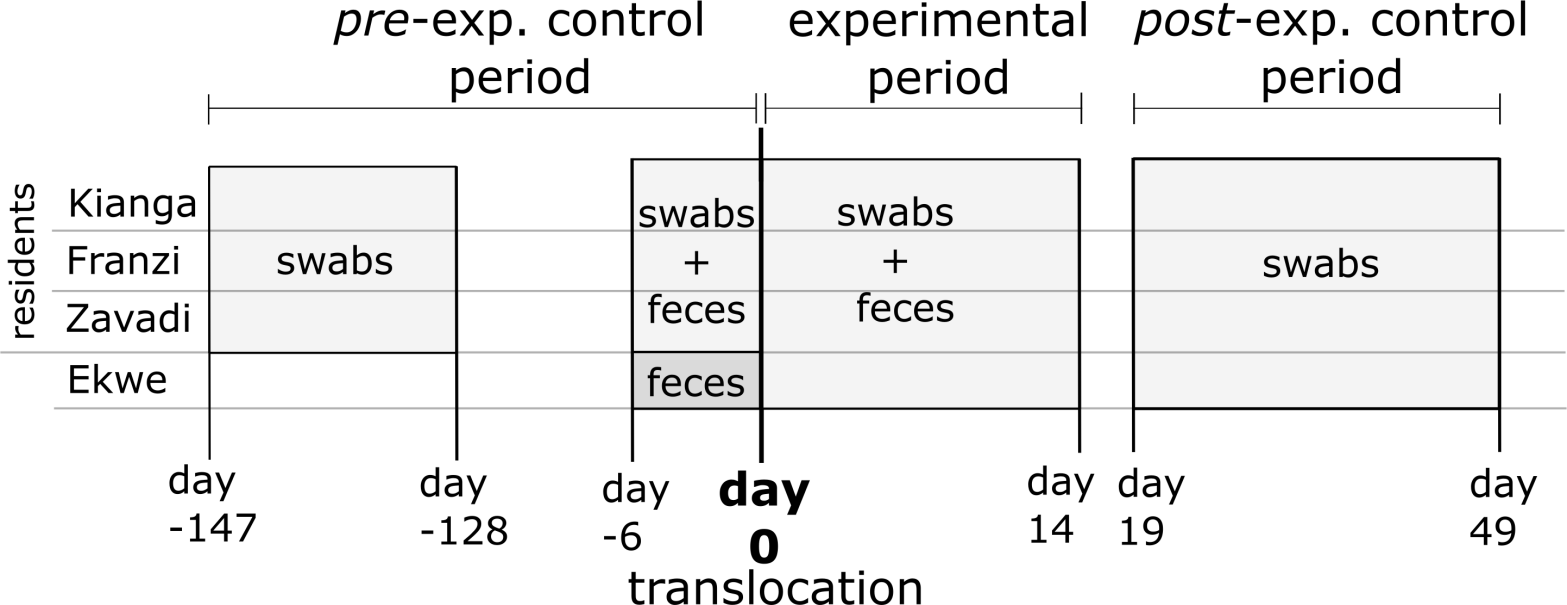
Experimental setup and sampling scheme. Day 0 indicates the day of the translocation; collected sample types are indicated in the grey-shaded boxes.

We assessed the frequency of sporadic EHV shedding in the zebras at Tierpark Berlin daily for 14 days post-translocation (termed ‘experimental period’), and in two control periods before and after the experimental period (Fig. 1). The pre-experimental control period comprised in total 25 days (19 days as a preliminary assessment four months before, and 6 days immediately before translocation). After the experimental period, we reduced the sampling frequency to two samples per week sampling. This post-experimental control period comprised 10 sampling days, from day 19 to 49 post-translocation.

### fGCM measurement

We collected one fecal sample per day from each of the four zebras at both locations over a time span from 6 days before, to 14 days after translocation (for detail see Fig. 1).

On the day of the arrival of the translocated individual only this animal was sampled, but not the resident ones, due to logistics. To control for unequal distribution of fGCM in the fecal boli, several subsamples were collected, pooled and homogenized. Samples were frozen immediately and stored at −80 °C until extraction.

We extracted 0.5 g wet weight (ww) per fecal sample (*N* = 81) with 4.5 ml methanol (90%) by automated shaking for 30 min. The extracts were then centrifuged at 1,000× g for 15 min, and 0.5 ml of the supernatant was diluted 1:1 with water for the subsequent Enzyme-Immunoassay (EIA). For fGCM concentration measurement we used an EIA with an antibody against 11 β-hydroxyetiocholanolone (Pribbenow et al., 2014).

### EHV shedding

Screening for EHV shedding was only conducted at Tierpark Berlin, where zebras were housed in an outdoor enclosure for most of the day, but were separated into individually assigned stalls for feeding of grain concentrate each morning. The animals were kept in their respective stalls for up to three hours. In order to trace shedding of EHV by nasal discharge, we collected swabs from each individual’s feed trough, on a daily basis. The swabs were subsequently screened for EHV DNA to detect viral shedding and genetically identify virus strains. During the pre-experimental control period (for 19 days, four months before the translocation event, and 6 days immediately before translocation), feed trough swabs of only the three resident zebras were collected. During the experimental period, swabs were collected from all four animals, including the translocated animal, for 14 days (apart from the day of the arrival of the translocated individual, due to logistical constraints). During the post-experimental control period (day 19 to 49) swabs were collected on 10 days from all animals. As the zebras were placed into individual stalls, feed troughs were only used by one respective animal. Before translocation, the mare at Zoo Mulhouse was exclusively housed in a group enclosure, therefore we were unable to collect swabs from surfaces which only this specific animal had access to. As an extension of the core study period we continued collecting trough swabs until day 49 post-translocation for all animals, under a reduced sampling scheme with sampling two days per week to screen for further EHV shedding. For swabbing, we used dry cotton swabs dipped in phosphate buffered saline. Feed troughs were cleaned daily after swab sampling, however a carry-over of viral DNA from one day to the next cannot be entirely excluded though we do note that carryover could not be pervasive as negative results were rather the norm than the exception. We collected two replicates of each swab sample to reduce the probability of false-negative results, which may be expected at a higher rate when samples are collected from the environment. Although it seems likely that our sampling approach will lead to an underestimation of the true shedding frequency, it can be expected that the observed frequency of shedding events correlates positively with the true frequency of viral shedding.

DNA was extracted from the swabs using a commercially available kit (NucleoSpin Tissue Kit, Macherey-Nagel, Düren, Germany) following the manufacturer’s instructions. A diagnostic herpesvirus nested PCR was performed as described in Kleiboeker et al. (2002) with modifications of the thermocycling protocol (Seeber et al., 2017). PCR products were visualized on a 1.5% agarose gel. Bands in the expected product size (225 bp) were excised from the gel and purified using a kit (NucleoSpin Gel and PCR clean-up; Macherey-Nagel, Düren, Germany), according to the manufacturer instructions. Purified PCR products were Sanger sequenced by LGC Genomics GmbH, Berlin, Germany.

All initial sequencing results exclusively matched equid gammaherpesviruses. However, the strain identification by BLAST alignment was often ambiguous due to the relatively short sequence length. In order to obtain larger fragments of the DNA polymerase gene we designed new PCR primers specific to equine gammaherpesviruses (expected size 707 to 710 base pairs): EGHf (5′-ATA GCC AAG ATA GCC AAG ATC C-3′), and EGHr (5′-GTG TCC CCG TAG ATG ACC TT-3′). We used the following PCR conditions: initial denaturation at 95 °C for 2 min, followed by 45 cycles of 95 °C (20 s), 61 °C (20 s) and 72 °C (45 s), and final elongation at 72 °C for 2 min. The EGHf primer was used for Sanger sequencing.

### Data analysis

To test the effect of presumed environmental stressors on fGCM levels we divided the core study period into four time periods, according to the management regime: (1) “before translocation”, (2) “separate phase 1”: after arrival of the new mare during which this individual was kept in a separate enclosure until day 4 post-translocation, (3) “common enclosure”: days 5 to 9 post-translocation, and (4) “separate phase 2”: during which the new mare was again kept in a separate enclosure, i.e., from days 10 post-translocation. The effect of presumed environmental stressors on fGCM concentrations was tested by fitting a general linear mixed model using the log-transformed fGCM concentration as the response variable. However, the gut passage time of feces is reported to be 28 ± 7 h in Grévy’s zebras (Steuer et al., 2011), whereas herpesvirus reactivation due to perceived stress may be a matter of minutes to only a few hours. Thus, to control for gut passage time we assigned each fGCM measurement to the previous day (referred to as time corrected fGCM concentrations), and fitted another general linear mixed model using time-corrected log-transformed fGCM concentrations as response variable.

To test for an increase in EHV shedding between the control periods and the experimental periods in the resident animals, we fitted two generalized linear mixed model with binominal errors. In the first model we analyzed the differences between the pre-experimental control period and the experimental period using data on the three resident individuals. In the second model we analyzed the differences between the post-experimental control period and the experimental period using data on all four individuals. As a response variable we used for each animal and study period the paired number of days with EHV shedding and days without shedding. As predictors we used in both models study period (control and experimental) as a fixed effect, and animal ID as a random effect.

To control for potential differences in the magnitude of the stress responses between the resident mares and the new mare we included residency status (“new” versus “resident”) as a predictor. In addition the respective time period (before, separate 1, common, separate 2) was used as a predictor variable. Furthermore, we included the interaction between residency status and time to test for the possibility that the time trajectory of the stress response might depend on residency status. Finally, to account for additional inter-individual variability and repeated sampling of individuals we included individual identity as a random factor in the model. Visual inspections indicated no obvious violations of assumptions of normality and homogeneity of error variances.

To investigate the relationship between fGCM concentrations and EHV-shedding, we fitted a generalized linear mixed model with the EHV shedding status as the response variable. As predictors we included the time-corrected log-transformed fGCM concentration as a fixed effect and individual identity as the random effect. Statistical analyses were performed in R version 3.2.5 (R Development Core Team, 2016), using the “glmmADMB” package (Fournier et al., 2012). The significance threshold was set at an *α*-level of *p* < 0.05.

Virus sequences were queried against GenBank (Benson et al., 2009) using BLAST. All viral sequences matched a single gammaherpesvirus strain (EHV-7) most closely and we could identify within strain variation. The aligned long EHV DNA polymerase (DPOL) gene sequences were used to determine a maximum likelihood tree with Asinine gammaherpesvirus 5 (AHV-5, accession no. FJ798319.1) as an out-group, using Geneious version 9.1.5 software (Kearse et al., 2012) and PhyML (Guindon et al., 2010). For comparative purposes, we included two respective EHV sequences which had been isolated from two of the study animals in a previous study outside of the core and extended sampling times of the current study (Seeber et al., 2017).

## Results

**Figure 2:**
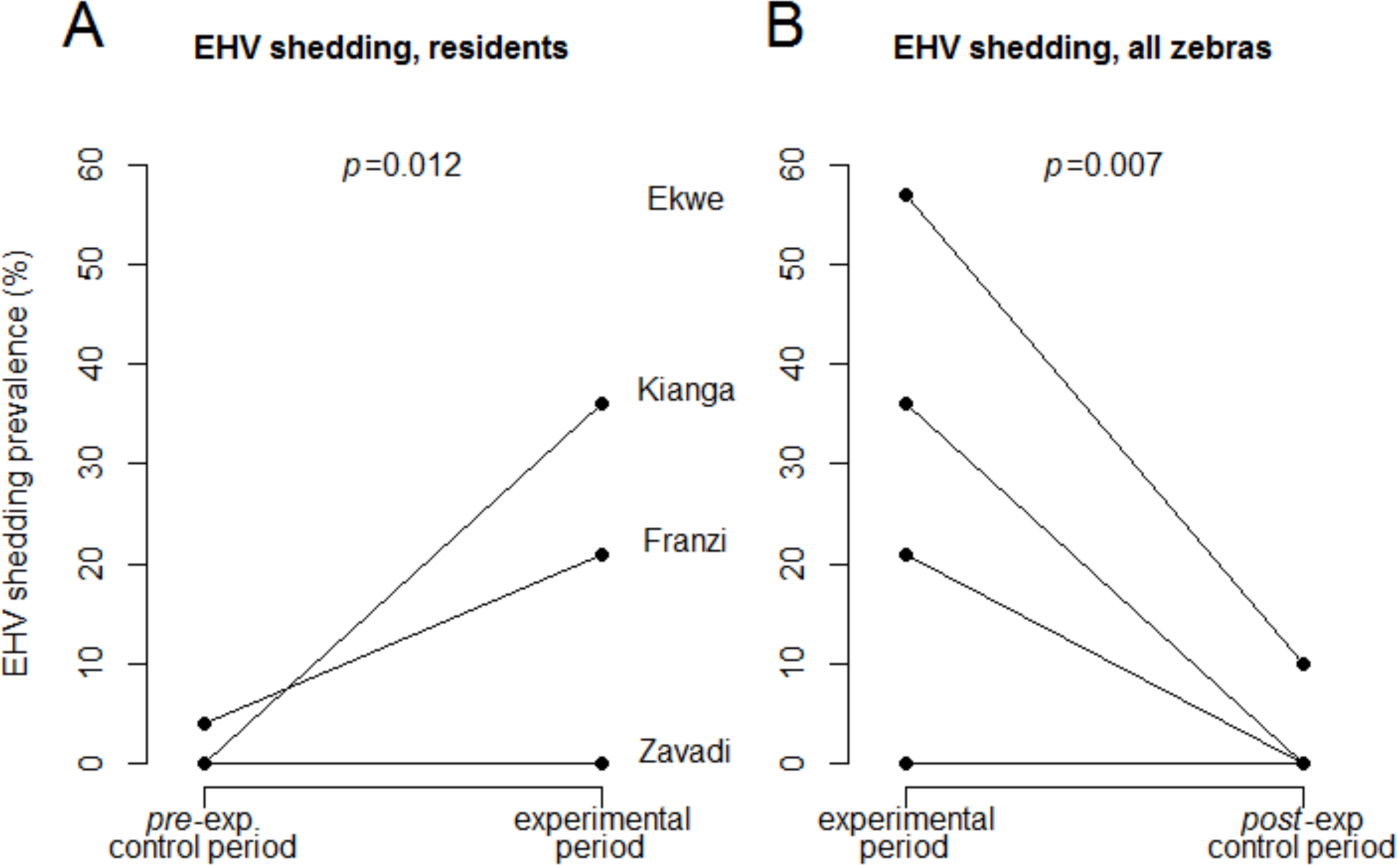
EHV shedding prevalence in the experimental and control periods. The relative prevalence of EHV shedding in the respective period (pre-experimental control period, experimental, and post-experimental control period) is shown (A) for the resident animals, and (B) for all animals.

In the resident zebras, the frequency of EHV shedding was elevated in the experimental period after the translocation event, compared to the pre-experimental period (*p* = 0.012; 95% CI [0.6–4.9], Fig. 2A). In all zebras, the frequency of EHV shedding was higher immediately after transport, than in the post-experimental control period (*p* = 0.007; 95% CI [0.79–5.09], Fig. 2B).

**Figure 3:**
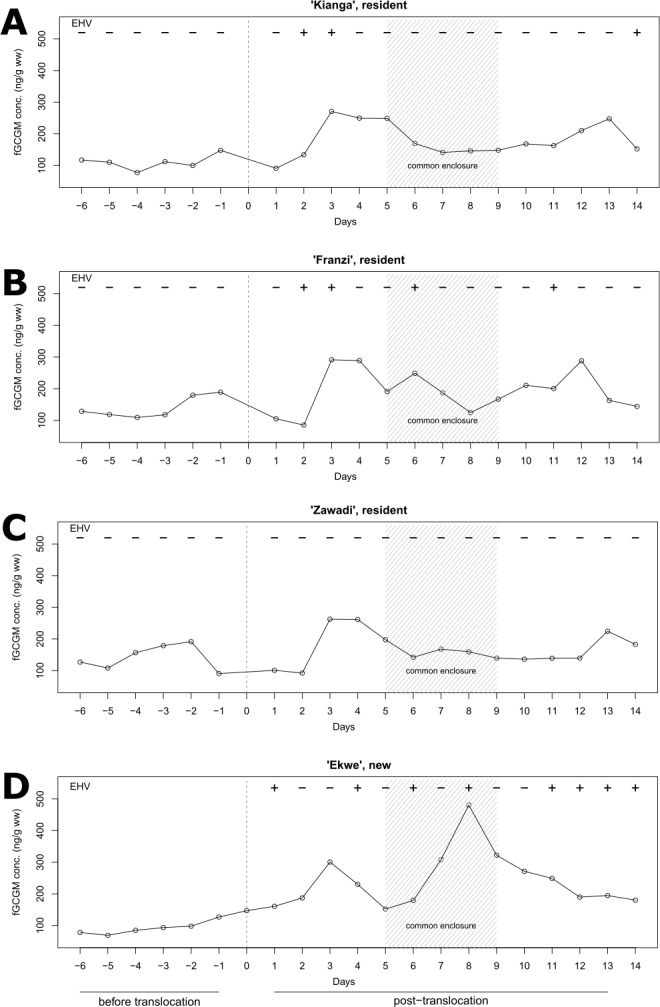
Individual fecal glucocorticoid metabolite (fGCM) and EHV shedding profiles of Grévy’s zebra mares. fGCM concentrations time-corrected by one day, from day -6 to day 13, and respective EHV shedding status (**+** EHV-positive; − EHV-negative) of each individual (A–D) with day 0 indicating the arrival of the translocated mare (D) at Tierpark Berlin. The shaded area (day 5 to day 9) indicates the time period when the four mares shared one common enclosure.

Furthermore, in all study animals, we found elevated fGCM concentrations in all time periods after the translocation event (Tables 1 and 2, Fig. 3). Residency status significantly affected fGCM concentrations only during the time spent in the common enclosure with higher concentrations in the translocated than in the resident mares (Tables 1 and 2, Fig. 3).

**Table 1:** Individual mean fecal glucocorticoid metabolite concentrations in captive Grévy’s zebra mares, per time period. Individual mean fGCM concentrations (ng/g ww) ± standard deviation in captive Grévy’s zebra mares, for each respective time period (before: 6 to 1 days before translocation event; separate 1: 0 to 4 days after translocation, separate enclosures; common: 5 to 9 days after translocation, common enclosure; separate 2: 10–14 days after translocation, separate enclosures).

**Individual**	**Residency status**	**Time phase**			
		Before translocation	Separate 1	Common	Separate 2
		**Mean fGCM concentrations (ng/g ww) ± std dev**			
*Kianga*	resident	110.8 ± 23.0	225.8 ± 62.0	154.5 ± 13.2	199.3 ± 44.2
*Franzi*	resident	140.3 ± 34.7	214.0 ± 97.9	187.4 ± 46.5	198.9 ± 64.0
*Zawadi*	resident	142.2 ± 40.1	203.5 ± 80.1	149.1 ± 14.0	171.4 ± 40.8
*Ekwe*	translocated	88.6 ± 18.2	196.2 ± 59.6	312.3 ± 109.3	203.3 ± 30.9

**Table 2:** Models on fGCM concentrations testing effects of time category and residency status. Results of the general linear model to compare fGCM concentrations between before translocation and the three post-translocation time periods, and the respective interaction with residency status. Statistically significant effects are highlighted in bold.

**Time span**	**Estimate**	**95% conf. int.**	**std. error**	*z*-value	***p***
Intercept	**4.78**	**[4.68, 4.88]**	**0.05**	**97.88**	<**0.001**
Residency status	−**0.27**	[−**0.41**, −**0.05**]	**0.09**	−**2.44**	**0.015**
Before translocation → separate 1	**0.66**	**[0.5, 0.82]**	**0.08**	**8.14**	<**0.001**
Separate 1: residency status	0.07	[−0.22, 0.35]	0.15	0.46	0.65
Before translocation → common enclosure	**0.28**	**[0.14, 0.43]**	**0.08**	**5.19**	<**0.001**
Residency status	**0.92**	[**0.63, 1.21**]	**0.15**	**6.23**	<**0.001**
Before translocation → separate 2	**0.46**	**[0.29, 0.64]**	**0.09**	**5.19**	<**0.001**
Residency status	0.22	[−0.12, 0.57]	0.18	1.26	0.21

**Figure 4:**
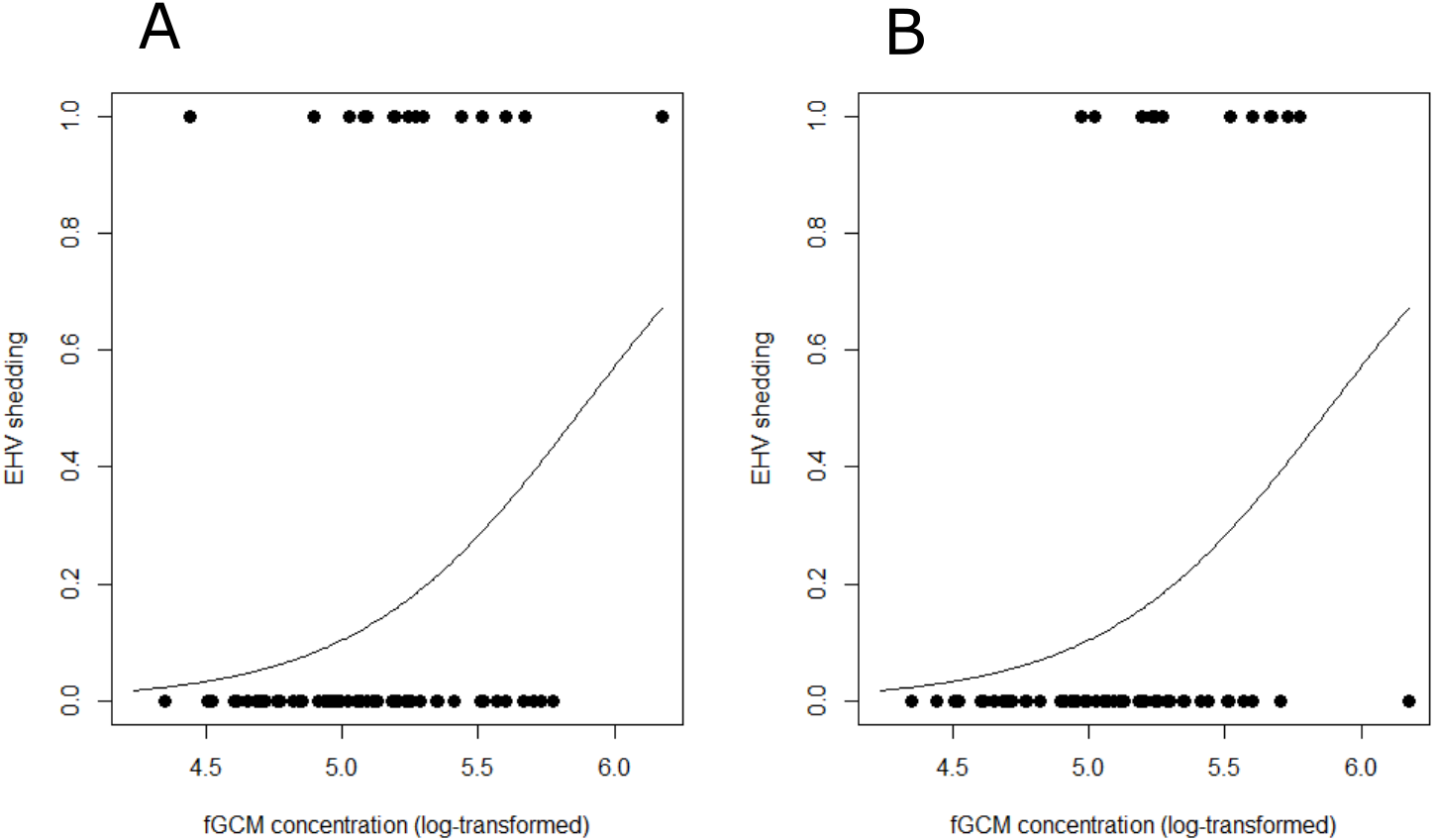
Relationship between fecal glucocorticoid metabolite concentrations (log-transformed) and detected EHV shedding. Each point represents an assessment of shedding for one individual on one day with a zero indicating that no shedding was detected and a one indicating that virus shedding was detected. The line shows the estimated increase in the probability of EHV shedding related to fGCM concentrations. (A) original values. (B) time-corrected values.

Based on data of all animals, we found no significant effect of individual fGCM concentrations on the likelihood of EHV shedding using the uncorrected values (*p* = 0.40; 95% CI [−1.08–2.71]; Fig. 4A). However, the model using the time-corrected values showed that an increase in fGCM concentrations significantly increased the likelihood of EHV shedding (*p* = 0.032; 95% CI [0.21–4.69]; Fig. 4B).

**Figure 5:**
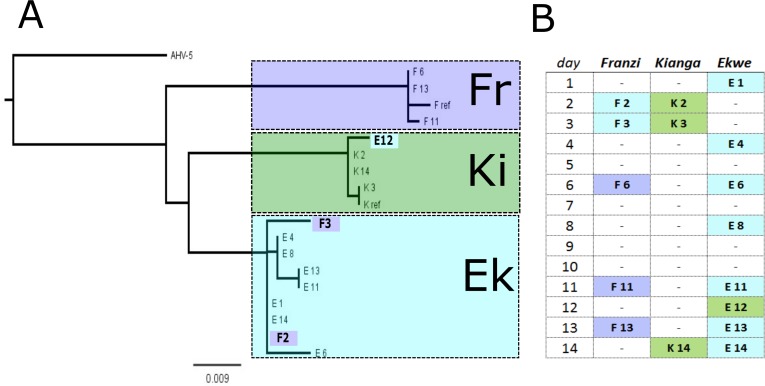
Phylogenetic relationships among detected EHV viruses. (A) Phylogenetic maximum likelihood tree generated from nucleotide sequences of a 600 bp fragment of the herpesvirus DPOL gene, using Asinine Herpesvirus 5 (AHV-5, accession no. FJ798319.1) as an out-group. Each tip represents one sample with the host individual’s initial letter and sampling day indicated. Two genotype variants in the clusters Fr and Ki had been identified in “Franzi” and “Kianga”, respectively, in a previous study, with the respective reference sequences labeled “F ref”, and “K ref”, respectively. Clusters “Fr”, “Ki”, and “Ek” are indicated by different frame lines. Colored tips indicate potential transmission events. (B) Genotypes as in Fig. 3. A indicated by frame lines, traced per individual during the two weeks after arrival of the new mare.

All virus strains detected belonged to the gamma-herpesvirinae, and all sequences most closely matched *Equus zebra*-Herpesvirus, and EHV-7 (with mostly equal identity scores, nucleotide identity 92–95%). We generated a maximum-likelihood phylogenetic tree, which indicated three genotype variants, including two genotype variants that had been identified in a previous study in the resident mares Franzi (EHV genotype “Fr”) and Kianga (EHV genotype “Ki”), respectively (Seeber et al., 2017) (Fig. 5A). Each variant was generally restricted to individual animals, apart from three instances: the strain first recovered from Kianga was retrieved once from Ekwe, and the strain initially recovered from Franzi was found twice in Ekwe (Fig. 5A). Throughout the entire study period, clinical signs of acute viral infection such as excessive nasal or ocular discharge, severe lethargy, or conjunctivitis were not observed in any of the study animals.

## Discussion

Three main results were generated by studying captive Grévy’s zebras: (1) the probability of EHV shedding was elevated in the experimental period immediately after the introduction of a new individual, (2) the group restructuring event may have acted as an environmental stressor that led to increased fGCM concentrations in both the resident and translocated animals, and (3) elevated GC levels correlated with EHV reactivation and virus shedding among the resident zebras. Thus, we provide evidence that stressors such as translocation and related changes in the social environment may elicit a physiological stress response in wild equids with subsequent EHV reactivation and potential virus transmission. Due to the lack of swab samples from the translocated zebra before transport, we cannot exclude that this animal was shedding EHV before the translocation event. However, in the resident animals the rate of EHV shedding increased significantly from the control to the experimental period, and, in all animals, decreased significantly from the experimental to the *post*-experimental control period. The small sample size of this study, however, may be a limiting factor for the interpretation of our results, and further investigation on a larger scale would be beneficial to confirm whether this is a general pattern in zebras. Ideally, an independent control group (e.g., individuals in a different enclosure) would be examined in order to comprehensively assess the effects of social stress.

Our results support the prediction that elevated GC levels correlate with EHV reactivation and virus shedding, using the time-corrected values. In contrast, the correlation with uncorrected time data did not reach statistical significance. Thus, our results indicate rapid EHV reactivation in the resident zebras in response to increased GC levels, and a time lag in the corresponding fGCM concentrations due to the gut passage. How fast such EHV reactivations typically occur in equids has, to our knowledge, not been investigated in detail. In a study of experimentally infected mice, herpesviruses reactivation could be traced to the earliest time point of screening 14 hours after exposure to a stressor (Sawtell & Thompson, 1992). However, the lack of earlier screening prevented a more detailed assessment, thus a more rapid virus reactivation may be possible. Taken together, our results are consistent with limited information in the literature indicating rapid herpesvirus reactivation in response to increased GC levels.

We have limited support for inter-individual EHV transmission: specifically, transmission seemed to have occurred from Ekwe to Franzi, and from Kianga to Ekwe (Fig. 5B). However, based on our results we cannot completely exclude the possibility that no transmission events occurred and instead all animals were already infected with the observed genotype variants before the onset of our study. The higher variability within the “Ek” genotype (up to 8 base pairs, Fig. S1 ) may indicate multiple closely related EHV variants shed by one individual co-infected host.

We expected that the long-distance transportation over 14 hours for an unsedated wild equid would be substantially more stressful than the subsequent integration into a new social group. Surprisingly, this expectation was not consistent with the temporal changes of fGCM concentrations that were observed in the translocated mare. In the first time period after arrival fGCM concentration peaked at 2.8-fold of the pre-translocation mean concentration, but during the time period in the common enclosure peaked at 4.7-fold (Fig. 3, Table 1). While we are not able to completely disentangle the role of transport as an independent stressor, it is likely that the additional increase in fGCM concentrations during the time period in the common enclosure can be attributed to factors of the social environment. In addition, the mean fGCM concentrations in the translocated mare increased from pre-transport to the time period immediately after transport by a roughly equal amount, as they did from this time period to the subsequent time period, in the common enclosure. Accordingly, in contrast to our expectation it appears that the integration into a new group was at least as stressful as the long distance transportation. However, due to the limited sample size in this study it remains unclear whether or not this is a general pattern. In this context it is important to consider that individual personality traits can affect how animals respond to challenging circumstances (termed coping style; e.g., (Baker et al., 2016; Cockrem, 2013)). Thus the adaptive capacity, and subsequently the physiological stress response in terms of GC secretion may be affected by individual stress perception and coping mechanisms. Our findings, even if tentative emphasize that social stress related to group restructuring can have major effects on physiological stress responses in wild equids.

Restructuring of social groups can be a source of considerable stress. For example, in horses, changes in group composition and consequent decreasing predictability of the social environment are reflected in increasing glucocorticoid levels (Alexander & Irvine, 1998; York & Schulte, 2014; Nuñez et al., 2014). Our results indicate that changes in group composition can elicit physiological stress responses in both newly introduced and resident Grévy’s zebras. Unexpectedly, the observed physiological stress responses occurred in the initial time period when animals were separated which prevented physical aggression between the residents and the new mare. Despite a physical barrier, introduction of a new mare to the environment was sufficient to elicit a stress response. During the subsequent time span in the common enclosure the new mare may have been exposed to actual physical aggression by the group of residents, as is common after group restructuring in horses (Alexander & Irvine, 1998), and would explain the further elevated fGCM concentrations in the new mare during this time span. Taken together our results show that changes in the social environment can be substantial stressors in captive Grévy’s zebra. However, because these animals live in artificially composed groups we cannot conclude that changes in group composition under more natural conditions elicit similarly strong physiological stress responses and with the subsequent reactivation of latent virus infections.

## Conclusions

Group restructuring affected the newly integrated mare and also all individuals indicating that not only stressors such as long-distance transport, but also less obvious stressors such as the perturbation of established social groups can potentially trigger viral reactivation. This result could have implications for the management of wild animals in captivity, which frequently includes exchange of breeding stock between zoological institutions. Subclinical virus shedding in equids exposed to stressors should be considered in order to reduce the risk of transmission to novel hosts, where EHV infection can have severe health consequences (Greenwood et al., 2012; Abdelgawad et al., 2014). However in practice, viral reactivation and transmission among equids might remain undetected due to the absence of clinical signs. Furthermore, environmental stressors may also play an important role in EHV reactivation and spreading in wild equid populations. For example, in free-ranging zebras elevated fGCM levels have been observed in the dry season when zebras aggregate in large herds (Cizauskas et al., 2015; Lea et al., 2017; Seeber et al., 2018). It could therefore be expected that EHV reactivation and transmission rates increase under such environmental conditions. However further research will be needed to fully understand the relationship between perceived stress, GC release and EHV reactivation in wild equids.

##  Supplemental Information

10.7717/peerj.5422/supp-1 Figure S1 Phylogenetic distances among detected EHV viruses Distance matrix showing the number of nucleotide differences among all pairs of the nodes in the phylogenetic tree shown in Fig. 3.

## Additional Information and Declarations

### Data Availability

The following information was supplied regarding data availability: Seeber, Peter A; Quintard, Benoît; Sicks, Florian; Dehnhard, Martin; Greenwood, Alex D; Franz, Mathias (2018): Fecal glucocorticoid metabolites in captive Grévys zebras, and acute shedding of equine Herpesvirus. PANGAEA, https://doi.org/10.1594/PANGAEA.888893.
