# Supplementary figures and images for: Environmental stressors may cause equine herpesvirus reactivation in captive Grévy’s zebras (Equus grevyi)
Source: PeerJ. 2018 Aug 22;6:e5422. doi: 10.7717/peerj.5422 (PMC6109370; doi:10.7717/peerj.5422)

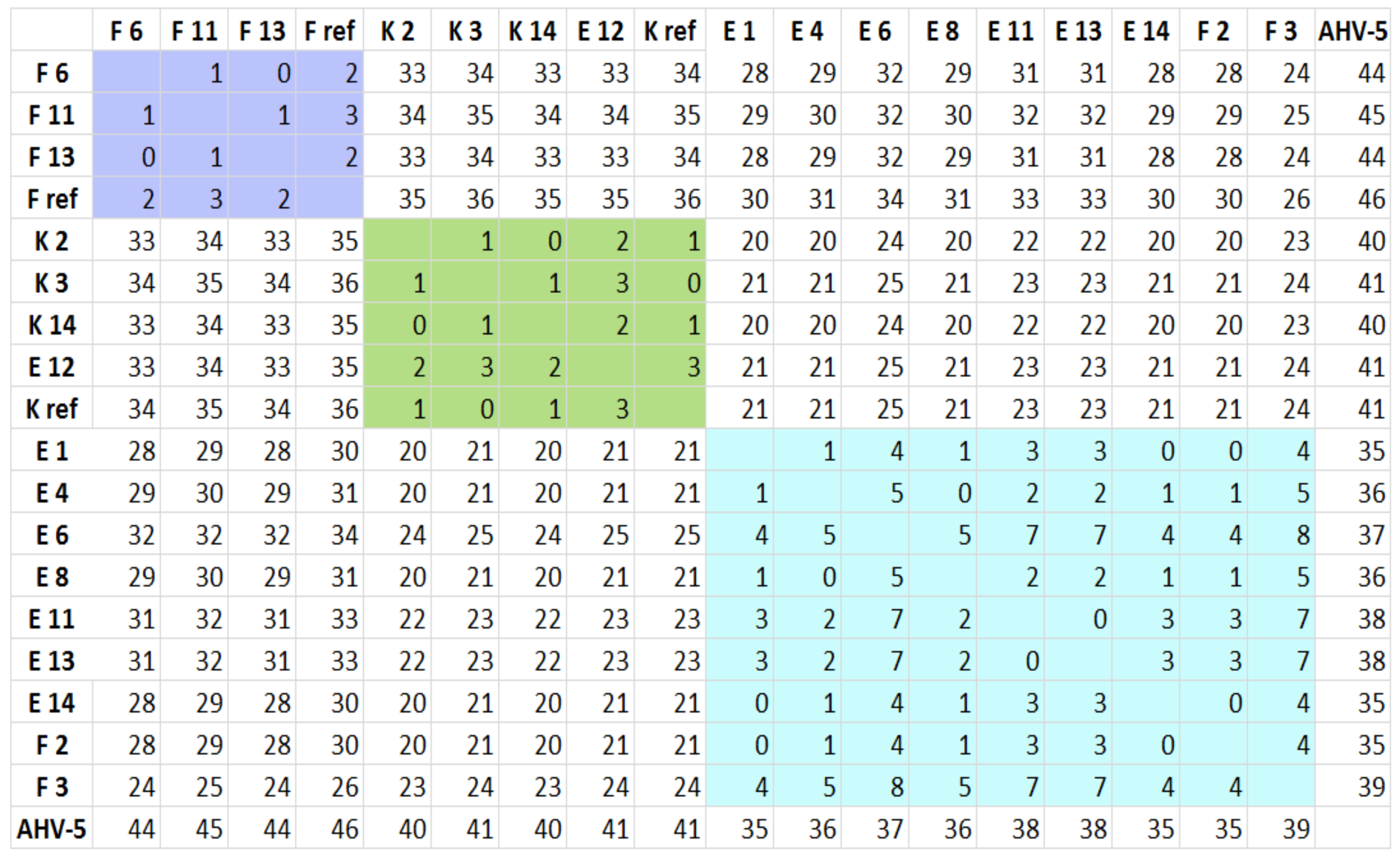

Supplement: Figure S1 — Distance matrix showing the number of nucleotide differences among all pairs of the nodes in the phylogenetic tree shown in Fig. 3. [file peerj-06-5422-s001.png]
